# On Urotropin

**Published:** 1904-05

**Authors:** 


### ABSTRACT.

### On Urotropin.

VINDEVOGEL, Laureate of the University of Brussels, 1897-1899, (Annals of the Royal Society of the Medical and Natural Sciences at Brussels, Vol. XI., Part 2, 1902,) aimed at determining whether and in what manner urotropin is decomposed in the organism into ammonium and formalin. The first tests which he made showed that the decomposition of urotropin is slight and slow in neutral cold solution (68 to 77° F.), and is more active when the temperature is raised, especially on ebullition. It is also more active in acid solution, even if cold.

From these facts it would seem that urotropin, when taken by mouth during digestion, is when it reaches the stomach in conditions favorable for the liberation of formalin, being in an acid medium at a temperature of 99.5° F. This is easily avoided, however, by administering the drug upon an empty stomach. Urotropin is then absorbed and circulated in the fluids of the body, the blood, lymph, and all alkaline or at least neutral media. It does not seem probable that formalin in quantity sufficient to give a reaction will be set free in them. But when the urotropin has been eliminated by the kidneys and mixes with the urine in the bladder, it is in an acid medium at a temperature of about 99.5° F., i. e., under conditions favorable for its decomposition and the liberation of formalin. It should therefore circulate in the blood as urotropin and should liberate formalin only after reaching the urinary bladder.

These considerations were borne out by animal experiments. When urotropin is administered subcutaneously to guinea-pigs, rabbits and dogs, urotropin, but no formaldehyde, is found in the blood. Formalin is found in the urine of most of these cases, but no satisfactory explanation was found for the exceptions to this rule. In healthy human beings the acidity of the urine is always markedly increased by urotropin. Formaldehyde is found in most of their urines, but here again there is no explanation for the exceptions.

When the urine is rapidly voided after excretion, it does not contain formalin. The demonstration of formalin is, however, easy, when the urine has remained three hours or more in the bladder. Hence urotropin in man is absorbed and circulates unchanged. It is eliminated by the kidneys unchanged, and the formalin is set free only in the bladder. From this last conclusion it follows that urotropin may be used to set free formalin in the urine, and that it will be found useful whenever it is necessary to effect vesical antisepsis.

Vindevogel then records 10 cases, in all of which the acidity of the urine, when of normal reaction, was markedly increased. and neutral and alkaline urines were acidified. Especially in chronic cystitis accompanying prostatic affections urotropin gave good results; there was a manifest amelioration of the most troublesome symptoms, the dysuria, pollakiuria, and the like. It always effects a rapid improvement followed by a complete cure in phosphaturia; success is more perfect, rapid and permanent than under the mineral acid treatment. Vindevogel believes also that it should render good service in cases of uric acid calculi. By keeping the urine acid and maintaining the phosphates in solution, it should prevent the increase in size of the uric acid calculus from the precipitation on it of phosphates.

